# Therapeutic Potential of the Gut Microbiota in the Prevention and Treatment of Sepsis

**DOI:** 10.3389/fimmu.2018.02042

**Published:** 2018-09-10

**Authors:** Bastiaan W. Haak, Hallie C. Prescott, W. Joost Wiersinga

**Affiliations:** ^1^Center for Experimental and Molecular Medicine, Amsterdam UMC, Amsterdam Infection & Immunity Institute, University of Amsterdam, Amsterdam, Netherlands; ^2^Department of Medicine, University of Michigan, Ann Arbor, MI, United States; ^3^VA Center for Clinical Management Research, Ann Arbor, MI, United States; ^4^Division of Infectious Diseases, Department of Medicine, Amsterdam UMC, Amsterdam Infection & Immunity Institute, University of Amsterdam, Amsterdam, Netherlands

**Keywords:** microbiota, sepsis, pathogenesis, therapeutics, probiotics and synbiotics, fecal microbiota transplantation

## Abstract

Alongside advances in understanding the pathophysiology of sepsis, there have been tremendous strides in understanding the pervasive role of the gut microbiota in systemic host resistance. In pre-clinical models, a diverse and balanced gut microbiota enhances host immunity to both enteric and systemic pathogens. Disturbance of this balance increases susceptibility to sepsis and sepsis-related organ dysfunction, while restoration of the gut microbiome is protective. Patients with sepsis have a profoundly distorted composition of the intestinal microbiota, but the impact and therapeutic potential of the microbiome is not well-established in human sepsis. Modulation of the microbiota consists of either resupplying the pool of beneficial microbes by administration of probiotics, improving the intestinal microenvironment to enhance the growth of beneficial species by dietary interventions and prebiotics, or by totally recolonizing the gut with a fecal microbiota transplantation (FMT). We propose that there are three potential opportunities to utilize these treatment modalities over the course of sepsis: to decrease sepsis incidence, to improve sepsis outcome, and to decrease late mortality after sepsis. Exploring these three avenues will provide insight into how disturbances of the microbiota can predispose to, or even perpetuate the dysregulated immune response associated with this syndrome, which in turn could be associated with improved sepsis management.

## Introduction

Sepsis is a highly heterogeneous and multifaceted syndrome that is caused by a dysregulated host response to an infection (1, 2). A disproportionate pro-inflammatory response to invasive infection was once believed to be the main pathophysiological paradigm of sepsis syndrome. However, recent studies have shown that patients with sepsis suffer from both sustained excessive inflammation and immune suppression, with a failure to return to normal homeostasis (3). Despite our increased understanding of the underlying pathophysiology of the syndrome, targeted therapies aimed at modifying the disrupted host response in patients with sepsis have been unsuccessful to date (4). Treating physicians are limited to supportive care and antibiotics as the disorder continues to drain billions of dollars and contributes to an estimated 5 million deaths worldwide each year (5, 6). Therefore, new tools in the management of sepsis are highly warranted.

In tandem with the advances of our understanding on the pathophysiology of sepsis, tremendous strides have been made in understanding the pervasive role of the gut microbiota in both health and disease states. Pre-clinical work has shown that a diverse and balanced gut microbiota is able to enhance host immunity to both enteric and systemic pathogens, and that disturbance of this balance potentially leads to increased susceptibility of sepsis (7, 8). Of interest, a handful of pioneering studies have shown that the composition of the intestinal microbiota is severely affected by sepsis, but the short- and long-term clinical consequences of these disturbances remain largely unknown (9–12). In this Review, we aim to provide an overview of the mechanisms through which the microbiota contributes to host defense in sepsis, and how these insights could be of relevance to this multi-facetted syndrome. Better insights into the drivers of microbiota-mediated host resistance could provide novel preventive and therapeutic strategies against sepsis.

## Microbiota-mediated host resistance

The intestinal microbiota is a complex ecosystem that consists of trillions of bacterial cells that have evolved with its hosts over millions of years (13). In the last decade, it has been revealed that these microbial communities are involved in a wide of array of functions, such as the digestion of food, production of hormones, and the development and maturation of the immune system (14–16). In addition, it has been shown that a state of disturbance of the intestinal microbiota, otherwise known as dysbiosis, plays an important role in increasing susceptibility to infectious diseases (17, 18). Several mechanisms have been uncovered on how the intestinal microbiota contributes to protection against enteric pathogens, such as *Clostridium difficile* (19). For example, intestinal microbes directly outcompete pathogens for nutrients, possess the capacity of producing antibacterial peptides, modify bile salts to render them harmful to other microorganisms, as well as drive increased mucus production and intestinal epithelial integrity. In addition, gut bacteria contribute to resistance to enteric pathogens by inducing the production of antibacterial factors by epithelial cells, and enhancing humoral responses against invading pathogens (19).

**Figure 1:**
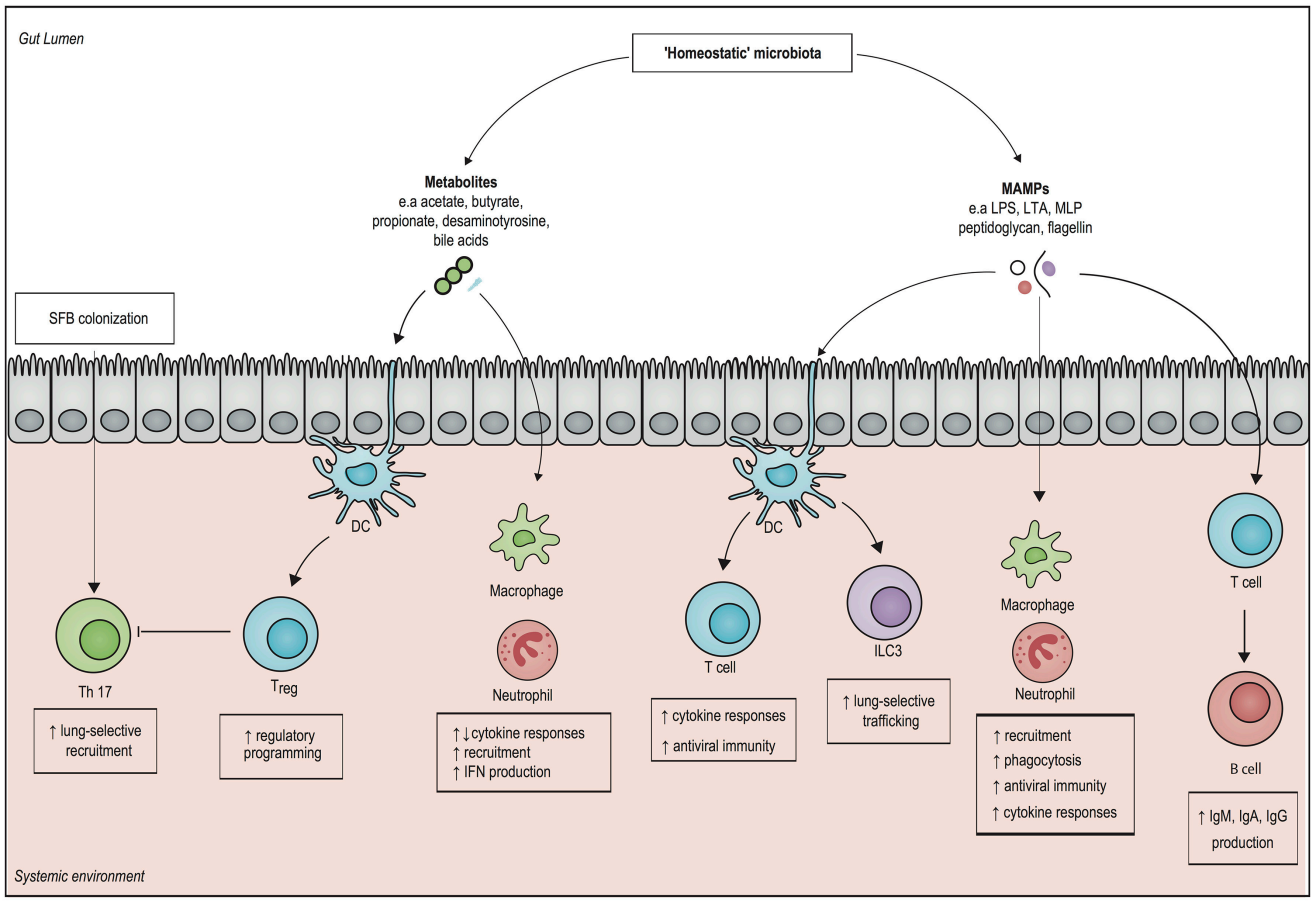
Overview of systemic immunomodulatory mechanisms associated with the microbiota. Structural components of gut microbiota, otherwise known as microbe-associated molecular patterns (MAMPs), can elicit a systemic pro-inflammatory response by activating pattern recognition receptors of both the innate and the adaptive immune system. Microbial metabolites, such as short chain fatty acids (SCFAs) modulate epigenetic changes in host leukocytes, which can induce both pro- and anti-inflammatory responses. The presence of the SCFAs butyrate and propionate drives the generation of regulatory T cells (T_reg_), which dampen inflammation. In addition, the gut metabolite desaminotyrosine enhances clearance of respiratory viruses by inducing type 1 interferon (IFN) responses. Direct interactions with epithelial cells by segmented filamentous bacteria (SFB) can enhance mucosal immunity by upregulating T helper 17 (Th 17) cells in both in the gut and in the lung. It is important to realize that our knowledge on microbiota-derived host-resistance is fragmented and it remains to be determined how these individual mechanisms fit in an overarching framework of systemic immunity. DC, dendritic cell; ILC3, type 3 innate lymphoid cell; Treg cell, regulatory T cell; IgA, Immunoglobulin A; IgG, Immunoglobulin G; IgM, immunoglobulin M; LPS, lipopolysaccharide; LTA, lipoteichoic acid; MLP, murein lipoprotein.

Recent insights have revealed that the microbiota also modulate systemic immunity (Figure 1). A 2016 hallmark study involving 500 healthy human volunteers linked the gut microbiota to inflammatory cytokine production capacity using *ex vivo* stimulation of whole blood and peripheral blood mononuclear cells: the observed inter-individual variation in cytokine responses was significantly correlated with the composition and function of the microbiota (20). Moreover, in healthy subjects in which the microbiota is disrupted by broad-spectrum antibiotics, systemic mononuclear cells produced lower levels of tumor necrosis factor (TNF)-α after *ex vivo* stimulation with lipopolysaccharide (LPS) (21). Of interest however, microbiota disruption by broad-spectrum antibiotics did not affect systemic innate immune responses in a human endotoxemia model, perhaps due to redundancies in the human immune response (22).

Numerous murine models have shown the existence of so called “gut-organ axes” such as the “gut-lung axis”, and the “gut-brain axis.” Besides cytokines, communication in these axes is probably mediated by microbe-associated molecular patterns (MAMPs), such as LPS, peptidoglycan and flagellin, as well as by microbiota-derived metabolites that are able to translocate from the gut into the systemic circulation, where they have the potential to modulate immune cells to enhance regulatory or pro-inflammatory responses (23, 24). In this way, intestinal bacteria can even direct the influx of immune effector cells into distant organs. For example, in newborn mice, exposure to gut bacteria increased homing of group 3 innate lymphoid cells (ILC3) from the gut mucosa toward the lung, which increased resistance against pneumonia (25). Other studies have shown that systemic exposure of microbiota-derived ligands increases the activity of alveolar macrophages and bone marrow derived neutrophils, which enhances the killing of Gram-positive and Gram-negative pathogens in the lung (26–28).

Similar priming mechanisms by toll-like receptor (TLR) ligands have shown to induce protection against respiratory viruses and fungi (29, 30). For example, neomycin-sensitive commensal bacteria induce the expression of messenger RNA (mRNA) for pro–IL-1β and pro–IL-18, which enhances the clearance of influenza virus in a T helper cell type 1, Cytotoxic T cell, and Immunoglobulin A (IgA) dependent manner (31). These findings were recently confirmed by Rosshart and colleagues, who developed a model of microbiota reconstitution from mice that were caught in the wild into genetically identical laboratory mice (32). Recolonization with a “natural” microbiota increased survival following influenza virus infection, which was attributed to a decreased pro-inflammatory response and a pronounced IL-10- and IL-13-mediated anti-inflammatory phenotype (32).

It has been shown that short-chain fatty acids (SCFAs), products of fiber fermentation by anaerobic bacteria, possess strong immunomodulatory properties through activation of certain G-protein-coupled receptors (GPRs), such as GPR41 and GPR43 (14, 33). In addition, the SCFAs butyrate and propionate facilitate the generation of extrathymic regulatory T cells, which have a key role in limiting inflammatory processes (34). Administration of butyrate also has the potential of restoring interleukin (IL)-10 levels in the lung by inhibiting histone deacetylase in myeloid-derived suppressor cells (MDSCs), which in turn reduces persistent lung inflammation during murine *K. pneumoniae* infection (35). Similar findings were observed in a cohort of allogeneic stem cell recipients, as a higher representation of butyrate-producing bacteria in the gut was associated with ameliorated respiratory viral infections (36). Besides short-chain fatty acids, it has also been found that desamonityrosine (DAT), a degradation product of flavonoids that is produced by *Flavonifractor plautii*, increases survival in mice infected with influenza through augmentation of type I interferon (IFN) signaling (37).

The immunomodulatory role of the microbiota extends beyond the lung, as presence of microbiota-derived LPS leads to increased recruitment of bone marrow-derived neutrophils, which enhances clearance of blood-borne pathogens, such as *Escherichia coli* and *K. pneumoniae* (38). Additionally, outer membrane components of Gram-negative bacteria in the gut, such as murein lipoprotein (MLP) and LPS, protect against experimental sepsis by modulating serum levels of Immunoglobulin G (IgG) and IgM, in a T cells and Toll-like receptor 4 on B cells-dependent manner (39, 40). These findings have recently been supplemented by Wilmore and colleagues (41), who have shown the that enriching the microbiota with members of the Proteobacteria phylum led to T cell-dependent increases in serum IgA levels by IgA-secreting plasma cells in the bone marrow. The resulting serum IgA bound specifically targeted a restricted number of pathogens that translocate from the gut into the systemic circulation, which increased resistance to sepsis (41). Despite the abovementioned indications that the microbiota is involved in the systemic host-defense against a wide variety of pathogens, further mechanistic studies are needed to provide overarching pathways that can be fitted into the framework of host-defense against sepsis.

## Causes and consequences of dysbiosis in sepsis

It has been shown that patients with sepsis have a profoundly distorted composition of the intestinal microbiota (9–12). In general, the diversity of the microbiota in patients with sepsis decreases rapidly upon hospital admission, a finding that becomes even more pronounced in later stages of their hospitalization (11). An extensive overview of the potential effects of sepsis on the intestinal microbiota has recently been published elsewhere (42). In short, these shifts in microbiota composition can be partially explained by clinical interventions, such as (par)enteral feeding, mechanical ventilation, as well as the omnipresent administration of proton pump inhibitors, opioids, vasopressors and—above all—antibiotics (42–44). Moreover, patients with sepsis have impaired gastrointestinal motility and diminished intestinal epithelial integrity, leading to a loss of “beneficial” anaerobic bacterial families, such as *Lachnospiraceae* and *Ruminococcaceae*, which further impairs intestinal epithelium function and allows for the expansion and potential translocation of aerobic opportunistic pathogens (45–47). The effects of sepsis on the microbiota extend beyond the gastrointestinal tract and can also influence the composition of for instance the lung and skin microbiota (42).

Several researchers have hypothesized that these shifts in microbiota composition potentially predispose patients to a state of immunosuppression (21, 48). There has also been renewed attention to the theory that systemic translocation of opportunistic gut bacteria increases the risk of organ failure (49). For example, the severity of acute kidney injury during sepsis can be ameliorated by administration of the three SCFAs acetate, propionate, and butyrate, which epigenetically attenuate the inflammatory process and reduce subsequent damage (50, 51). In addition, reconstitution of mice with the commensal *E. coli* O21:H+ induces signaling of the IGF1/PI3K/Akt pathway, which in turn prevents muscle wasting triggered by sepsis (52). Studies from the previous century showed that translocation of intestinal bacteria seems to be also associated with the development of acute respiratory distress syndrome (ARDS) (53, 54). Dickson and colleagues have recently found support for these findings by revealing that during murine sepsis and in human patients with ARDS, lung communities are enriched by bacteria that have translocated from the intestine. The presence of these communities, such as *Bacteroides spp*, is correlated with the intensity of systemic inflammation (55). Finally, preliminary research in mice and patients who died of sepsis suggests that bacterial translocation could also be associated with acute neuro-inflammation in sepsis (56), which aligns with the notion that gut microbes play a role in regulating the central nervous system (57). These studies provide clues that the extreme shifts observed in sepsis patients are not merely markers of illness severity, but also potentially contribute to worse outcome. However, human translational studies are lacking, so our current understanding of the short- and long-term consequences of ICU-related dysbiosis in clinical practice is limited (42). Further studies are needed to confirm that disturbances of the microbiota truly contribute to organ failure, and that dysbiosis is not merely a reflection of the profound dysregulation that is present during sepsis.

## Potential for modulation of the microbiota in the management of sepsis

Harnessing the immunomodulatory properties of the microbiota could provide an attractive preventive and therapeutic opportunity for sepsis. Modulation of the microbiota has been studied extensively outside of the sepsis arena, and predominantly consists of either resupplying the pool of beneficial microbes by administration of probiotics, improving the intestinal microenvironment to enhance the growth of beneficial species by dietary interventions and prebiotics, or by totally recolonizing the gut with a fecal microbiota transplantation (FMT). We propose that, within the course of sepsis, three therapeutic approaches exist that, when exploited, could potentially improve prevention and management of the disorder.

### Avenue 1: targeted microbiota modulation for at-risk patients, prior to sepsis development

The abovementioned pre-clinical observations demonstrating a link between dysbiosis and increased risk of sepsis have now gained some preliminary footing in human studies. For example, patients undergoing allo-HCT who develop antibiotic-induced dysbiosis have a 5- to 9-fold increased risk of bloodstream infection and sepsis (58). In line with these findings, a retrospective cohort of over 10.000 elderly patients in the United States showed that hypothesized instances of dysbiosis were associated with a more than 3-fold increased incidence of a subsequent hospitalization for sepsis (7). And, expanding on these findings, Baggs et al. recently showed that exposure to longer durations of antibiotics, additional classes of antibiotics, and broader-spectrum antibiotics during hospitalization are each associated with dose-dependent increases in the risk of subsequent sepsis (59). This association was not found for other causes of hospital readmissions, suggesting that the association between antibiotic exposure and subsequent sepsis is related to microbiome depletion, not merely illness severity (59).

Probiotics have been evaluated for preventing nosocomial infection in many smaller studies, and meta-analyses suggest that probiotics are safe and effective at preventing infection in both post-operative and mechanically ventilated patients (60, 61). However, the small size of the individual studies, as well as the variable type and dose of probiotic therapy limit strong conclusions. A large-scale study of probiotics is underway in Canada to test the benefit of probiotics for preventing ventilator-associated pneumonia (clinicaltrials.gov: NCT02462590). Excitingly, in a recent randomized, double blind, placebo-controlled trial of 4,556 healthy, term infants in rural India, administration of an oral synbiotic preparation (*Lactobacillus plantarum* plus a fructooligosaccharide) resulted in a 40% relative risk reduction for lower respiratory tract infections, sepsis, and death (62). The study was stopped early due to overwhelming benefit, and the authors have asserted that with a single investment of only 27 dollars, one case of neonatal sepsis could be prevented, which would be an unparalleled breakthrough in decreasing the worldwide incidence of sepsis. However, other studies that assessed the role of probiotics in other populations, such as pre-term and underweight children, showed no differences in sepsis incidence and mortality, indicating that the potential effects of microbiota restoration are not uniformly conserved across populations and settings (63, 64). Therefore, large stratified cohorts that collect data before the potential onset of sepsis are warranted to help pinpoint in which context gut commensals are able drive protection against sepsis. Recognizing these drivers of protection could help develop means of targeted microbiota restoration in order to decrease the incidence of sepsis.

### Avenue 2: addressing dysbiosis during the course of sepsis

Based on the abovementioned findings that probiotics and synbiotics decrease the incidence of infections complications at the ICU, a strong interest exists to investigate the use of these agents to improve outcome in patients with sepsis (60, 61). However, implementation of probiotic treatment in sepsis has been limited due to a lack of consensus with regard to the optimal choice of probiotic species and dosages, as well as lingering concerns for patient safety (65, 66). Recently, FMT has been used successfully in four cases of therapy-resistant sepsis and diarrhea (67–69). These initial studies describe that recolonization of the intestinal microbiota via FMT during sepsis could counterbalance dysbiosis, induce recovery of gut microbial barrier, which in turn could potentially improves the outcome of sepsis on the ICU. However, the first clinical studies investigating this modality on a larger scale have yet to commence. Other studies propose that treatment with microbiota-targeted metabolites, such as butyrate or other SCFAs, could be used as more stable immunomodulators than their bacterial counterparts (70). However, at the moment, surprisingly few studies have investigated the use of microbiota-targeted therapies during sepsis (45). These studies are essential to define the efficacy, safety and potential benefit of these therapies in the management of the disease.

### Avenue 3: rapid microbiota restoration in sepsis survivors

**Figure 2:**
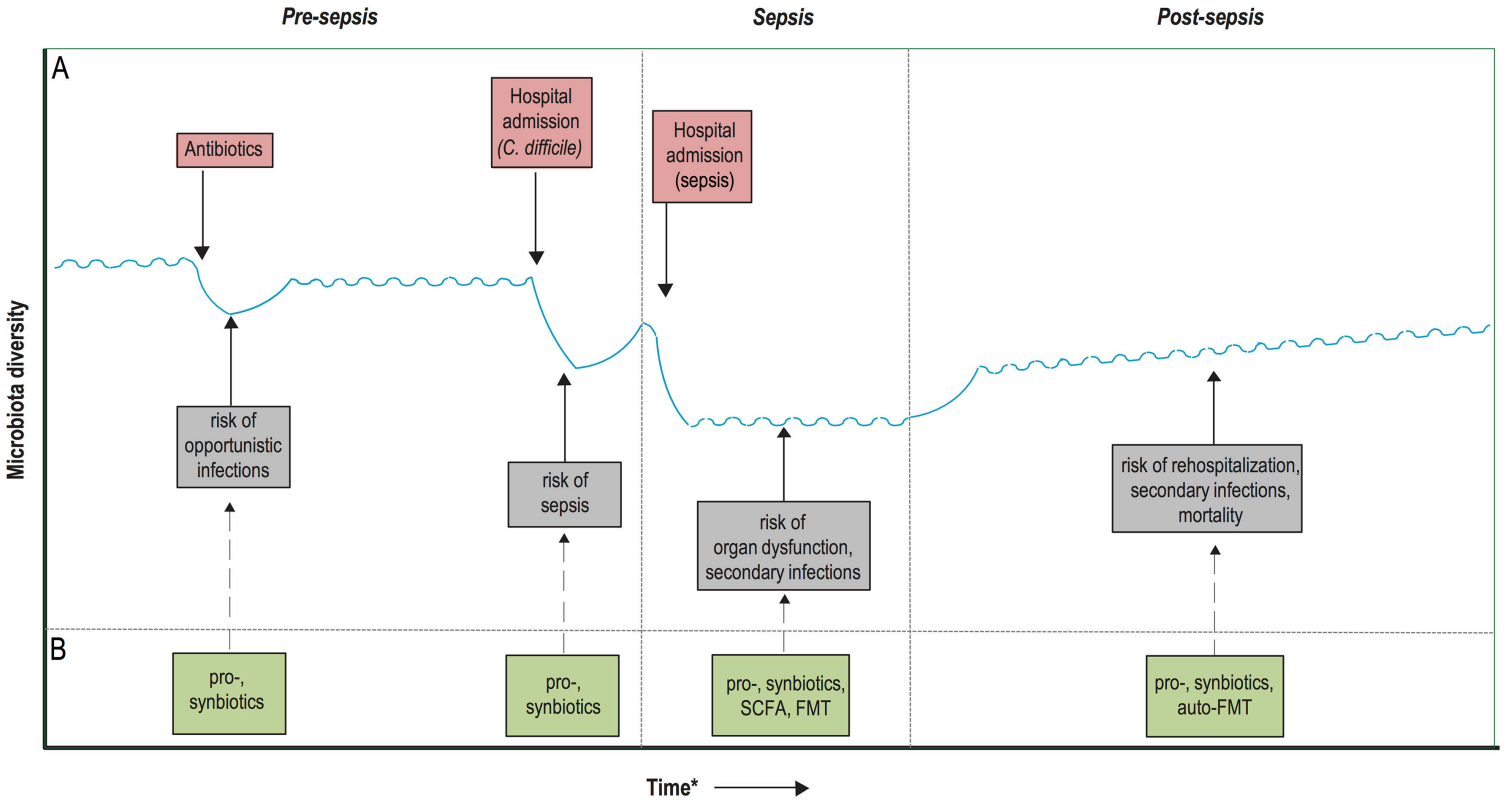
Timeline of potential microbiota-associated interventions prior to, during and post-sepsis. **(A)** Hypothetical diagram of the associations between the risk of dysbiosis and sepsis development and outcome. Dysbiosis occurs due to the administration of antibiotics and/or hospitalizations, recover quickly upon hospital discharge or cessation of antibiotic treatment, but it often takes long for the microbiota to recover entirely. These periods of dysbiosis predispose to the development of opportunistic infections, which in turn further worsens the state of dysbiosis and predispose to sepsis development. During sepsis, the microbiota composition is extremely hampered, which has been associated with an increased risk of secondary infections, immunosuppression and potentially even organ dysfunction. Recolonization of a homeostatic microbiota is slow upon hospital discharge and sepsis recovery, which might contribute to prolonged immunosuppression, rehospitalization due to infections and increased mortality. **(B)** Probiotics and synbiotics have the potential to enhance microbiota colonization in neonates, as well as accelerate the recovery of the microbiota after periods of dysbiosis, which in turn could provide protection against the development of sepsis. During the course of sepsis, microbiota disruption could be ameliorated by the administration of pro- and synbiotics, which potentially reduces the occurrence of secondary infections. In addition, future treatment with fecal microbiota transplantation (FMT) or targeted restoration with microbiota-associated metabolites, such as short-chain fatty acids (SCFAs), could reduce the risk of prolonged immunosuppression and organ dysfunction. Finally, microbiota restoration after sepsis recovery could be accelerated with pro- and synbiotics, or by an autologous FMT. ^*^Time depiction is schematic.

Pre-clinical models have shown that a state of immunosuppression can be maintained long after recovery from sepsis (71, 72), which is thought to predispose sepsis survivors to infections and increased mortality up to 2 years following sepsis discharge (73–75). Indeed, recent studies have shown that 20% of the patients who survive sepsis has a late death—predominantly caused by infections, respiratory failure, and aspiration pneumonitis—that cannot be attributed to prior health status and comorbidities (76). These findings have two implications. First, the general dysregulation that occurs during sepsis is profound and its consequences are long lasting. Second, as late death after sepsis cannot be attributed to previous health status, it suggests that targeted interventions can be designed to reverse this hypothesized state of immunosuppression and decrease mortality after sepsis (76). Given the potential role of dysbiosis in maintaining immunosuppression, restoration of gut commensals after sepsis discharge by probiotic administration in order to reduce late infections and subsequent mortality warrants further research. Another concept within the scope of microbiota restoration after sepsis discharge consists of microbiota auto banking, which encompasses the storage of feces of patients in an ambulatory setting (77). These samples could subsequently be used in an autologous FMT in order to restore the microbiota after periods of dysbiosis, in this case after sepsis discharge. Of interest, the efficacy of auto-FMT for the prevention of infections after a period of dysbiosis is currently studied within a cohort of patients receiving allo-HCT (clinicaltrials.gov: NCT02269150). A schematic overview of the proposed therapeutic avenues in the prevention and management of sepsis is depicted in Figure 2.

## The road ahead

Our understanding on how disturbances of the microbiota can predispose to, or even perpetuate the dysregulated immune response associated with sepsis is fragmented. The more we know about these collections of micro-organisms that inhabit our body compartments, the more we realize how delicate the mechanisms are through which these microbes contribute to homeostasis. Moreover, micro-organisms that reside in other body compartments than the gut, such as the skin and the lung, are likely to play an equally important but underexplored role in baseline immune function (42, 78, 79). To add an additional layer of complexity; emerging data indicate that eukaryotic viruses regulate, and are in turn regulated by the host and other microbial constituents that inhabit the intestine (80, 81). These co-inhabitants consist not only of bacteria, but also bacteriophages, helminths, and fungi, which influence each other through a series of processes termed “transkingdom interactions.” This holistic view of the human microbiota represents an uncharted paradigm that will need to be further evaluated in the context of sepsis.

In general, large human cohort studies that document microbiota composition, prior to, during and after an episode of sepsis are needed to identify those commensals that protect against sepsis vs. those that are potentially associated with increased susceptibility and worse outcome. These insights could allow us to identify bacterial groups that are associated with immunological resilience, which could be harnessed as potential biomarkers of susceptibility to sepsis or adverse outcome. In parallel, mechanistic animal studies—that should include the use of older and “dirtier” mice (32, 82, 83) and better mimic the clinical scenario by using antibiotics and other treatments often given in sepsis (84)—are needed to disentangle potential mechanisms that drive the phenotypes seen in the human situation. These combined efforts have recently lead to the successful identification and development of new next-generation probiotics that are able to selectively treat specific pathogens, such as *C. difficile* and vancomycin resistant *Enterococcus* (VRE) (85, 86). Despite the significant challenges ahead, we foresee that further implementation of microbiota-targeted therapies could improve sepsis management and prevention.

## Disclosures

The views expressed in this article are those of the authors and do not necessarily reflect the position or policy of the Department of Veterans Affairs or the US government.

## Author contributions

BH, WW, and HP equally contributed to the write-up of the manuscript. BH drafted the figures and performed the literature search.

### Conflict of interest statement

The authors declare that the research was conducted in the absence of any commercial or financial relationships that could be construed as a potential conflict of interest.
